# *Hemidesmus indicus* induces apoptosis via proteasome inhibition and generation of reactive oxygen species

**DOI:** 10.1038/s41598-019-43609-5

**Published:** 2019-05-10

**Authors:** Eleonora Turrini, Elena Catanzaro, Lorenzo Ferruzzi, Alessandra Guerrini, Massimo Tacchini, Gianni Sacchetti, Guglielmo Paganetto, Francesca Maffei, Valentina Pellicioni, Ferruccio Poli, Patrizia Hrelia, Manuela Mandrone, Piero Sestili, Maurizio Brigotti, Carmela Fimognari

**Affiliations:** 1 Department for Life Quality Studies, University of Bologna, Rimini, Italy; 2 Department of Life Sciences and Biotechnology, University of Ferrara, Malborghetto di Boara, Ferrara, Italy; 3 Department of Pharmacy and Biotechnology, University of Bologna, Bologna, Italy; 4 Department of Biomolecular Sciences, University of Urbino “Carlo Bo”, Urbino, Italy; 5 Department of Experimental, Diagnostic and Specialty Medicine, University of Bologna, Bologna, Italy

**Keywords:** Chemotherapy, Leukaemia

## Abstract

Proteasome inhibition represents an important anticancer strategy. Here, we studied the mechanisms at the basis of the pro-apoptotic activity of the standardized decoction of *Hemidesmus indicus*, a plant evoking a complex anticancer activity, and explored its inhibition of proteasome activity in human leukemia cells. Additionally, we preliminary tested the cytotoxicity of some *H*. *indicus*’s phytochemicals on leukemia cells and their intestinal absorption on a human intestinal epithelium model consisting of a monolayer of differentiated Caco2 cells. We observed a potent antileukemic effect for *H*. *indicus*, imputable to the modulation of different critical targets at protein and mRNA levels and the reduction of the 26S proteasome expression. We found that some phytomarkers of *H*. *indicus* decoction passed through the enterocyte monolayer. Overall, our study supports the pharmacological potential of *H*. *indicus*, which can represent an interesting botanical drug in the oncological area.

## Introduction

The ubiquitin-proteasome system is responsible for the degradation of intracellular regulatory proteins and the maintenance of cellular protein homeostasis^1^. This system affects signaling pathways involved in cell-cycle regulation, apoptosis, and stress response. Cancer cells generally lack cell-cycle checkpoints and may be exceptionally vulnerable to the stress imposed by proteasome inhibitors^2^, which can represent an important anticancer strategy.

Bortezomib is a specific and selective inhibitor of 26S proteasome, approved for the treatment of patients with multiple myeloma and mantle cell lymphoma^3^. However, its use is limited by its dose-limiting toxicity characterized by a painful sensory neuropathy^4^. Thus, the definition of strategies for containing the toxicity of bortezomib or the identification of products with a better pharmaco-toxicological profile is crucial for the treatment of patients. An approach could be the co-administration of bortezomib with drugs increasing its antitumor activity leading to a reduction of the recommended doses and its dose-dependent toxicity^5^. Moreover, several second-generation proteasome inhibitors are now under clinical development for cancer therapy^6^.

Over the past several decades, natural products have been explored as proteasome inhibitors. Among them, salinosporamide A, a marine-derived product, currently is in clinical trials for the treatment of multiple myeloma^7^. Terrestrial derivatives including lactacystin, ubistatins, epoxomicins, and green tea polyphenols^8^ induce cancer cell death via the ubiquitin-proteasome pathway. Most of them are in the pre-clinical phase, with the exception of green tea polyphenols, in phase II-trial, and the epoxomicin derivative carfilzomib, approved for treatment of multiple myeloma patients^9^.

Many studies report the anticancer, antiarthritic, antimicrobial, antiulcer, immunomodulatory activities of *Hemidesmus indicus* (HI, *Apocynaceae*)^10^. Its complex nature of botanical drug confers HI the ability to interact simultaneously with different biological targets, making it a valuable candidate to contrast the biological complexity of multifaceted diseases, such as cancer. Furthermore, the growing studies on its anticancer effects disclose an emerging complexity of its anticancer mechanisms. As an example, the root decoction of HI is able to prompt all *in vitro* hallmarks of a particular type of cell death, i.e. the immunogenic cell death^11^, which activates dendritic cells to promote antitumor immunity^12^. A clinically relevant evidence is that it exhibits cytotoxic effect in blasts from acute myeloid leukemic patients and potentiates the cytotoxicity of widely used anticancer drugs^13^. A recent study also reported that HI decoction inhibits the angiogenic cascade and decreases microvessels formation in both normoxia and hypoxia^14^.

Considering those observations, here we evaluated the mechanisms at the basis of the pro-apoptotic activity of HI decoction and explored its inhibition of proteasome activity in human leukemia cells. Additionally, we preliminary tested the cytotoxicity of some HI phytochemicals on leukemia cells and their intestinal absorption on Caco2 cell monolayer mimicking the human intestinal epithelium treated with the decoction.

## Results

### Chemical composition of HI decoction

The HI decoction was first separated into two parts: hydrophilic (the most polar, which contained mainly sugars) and lipophilic (the most apolar). The latter fraction was subsequently separated through silica gel, and eight main fractions were collected (fraction 1, 2, 3, 4, 5, 6, 7, and 8) (Table 1). The analysis performed by GC-MS allowed identifying the main phytochemicals that characterize each fraction. Due to the lipophilic characteristics of the solvent system, the extracted molecules were mainly triterpenoids.

**Table 1:** Compounds detected in the eight fractions of HI decoction.

Fractions	Compounds detected*
1	α-amyrin, lupeol, β-amyrin acetate, α-amyrin acetate and lupeol acetate
2	3-hydroxy-4-methoxybenzaldehyde, 2-hydroxy-4-methoxybenzaldehyde, β-amyrin, α-amyrin, lupeol, β-amyrin acetate, α-amyrin acetate and lupeol acetate
3	C3-hydroxy-4-methoxybenzaldehyde, C2-hydroxy-4-methoxybenzaldehyde, α-amyrin, lupeol, β-amyrin acetate, α-amyrin acetate and lupeol acetate
4	3-hydroxy-4-methoxybenzaldehyde, 2-hydroxy-4-methoxybenzaldehyde, α-amyrin, lupeol, β-amyrin acetate, α-amyrin acetate and lupeol acetate
5	t3-hydroxy-4-methoxybenzaldehyde, t2-hydroxy-4-methoxybenzaldehyde, Cα-amyrin, lupeol, β-amyrin acetate, Cα-amyrin acetate and lupeol acetate
6	tβ-amyrin acetate, α-amyrin acetate
7	tβ-amyrin acetate, α-amyrin acetate, Cβ-sitosterol
8	2-hydroxy-4-methoxybenzoic acid and sugars

* In order of elution; C: most characterizing molecule of the fraction; t: found in traces.

α-Amyrin, lupeol, β-amyrin acetate, α-amyrin acetate, and lupeol acetate, in this order of elution, characterized fraction 1. The relative abundance of these molecules changed during the elution. However, in the fraction 1 β-amyrin acetate was at the highest level compared to the other fractions. Even if we detected the above-mentioned molecules in each fraction, their presence gradually decreased until fraction 7, where we observed just β-amyrin acetate and α-amyrin acetate (Table 1).

In addition to the previous compounds, fraction 2 showed the presence of β-amyrin and two vanillin derivatives: 3-hydroxy-4-methoxybenzaldehyde (3,4A) and 2-hydroxy-4-methoxybenzaldehyde (2,4A), which represent the most characterizing molecules of fraction 3 along with vanillin itself.

The presence of the aldehydes decreased in the fraction 4, where the triterpenoids become the main molecules. α-Amyrin and α-amyrin acetate co-eluted respectively with lupeol and lupeol acetate. In fraction 4, they showed a higher relative level compared to the other terpenoids.

In the fraction 5, the presence of 3,4A, 2,4A, lupeol acetate, lupeol, and β-amyrin acetate decreased. Thus, this fraction seemed constituted mainly by α-amyrin and α-amyrin acetate.

α-Amyrin acetate, traces of aldehydes, and β-amyrin acetate characterized fraction 6, where we did not observe the presence of lupeol, α-amyrin, and lupeol acetate.

In the fraction 7, the GC-MS analyses highlighted the presence of β-sitosterol and traces of β-amyrin acetate.

2-Hydroxy-4-methoxybenzoic acid (2,4Acid) and a minority of sugar components characterized fraction 8.

### The fractions of HI decoction decrease cell viability

**Figure 1:**
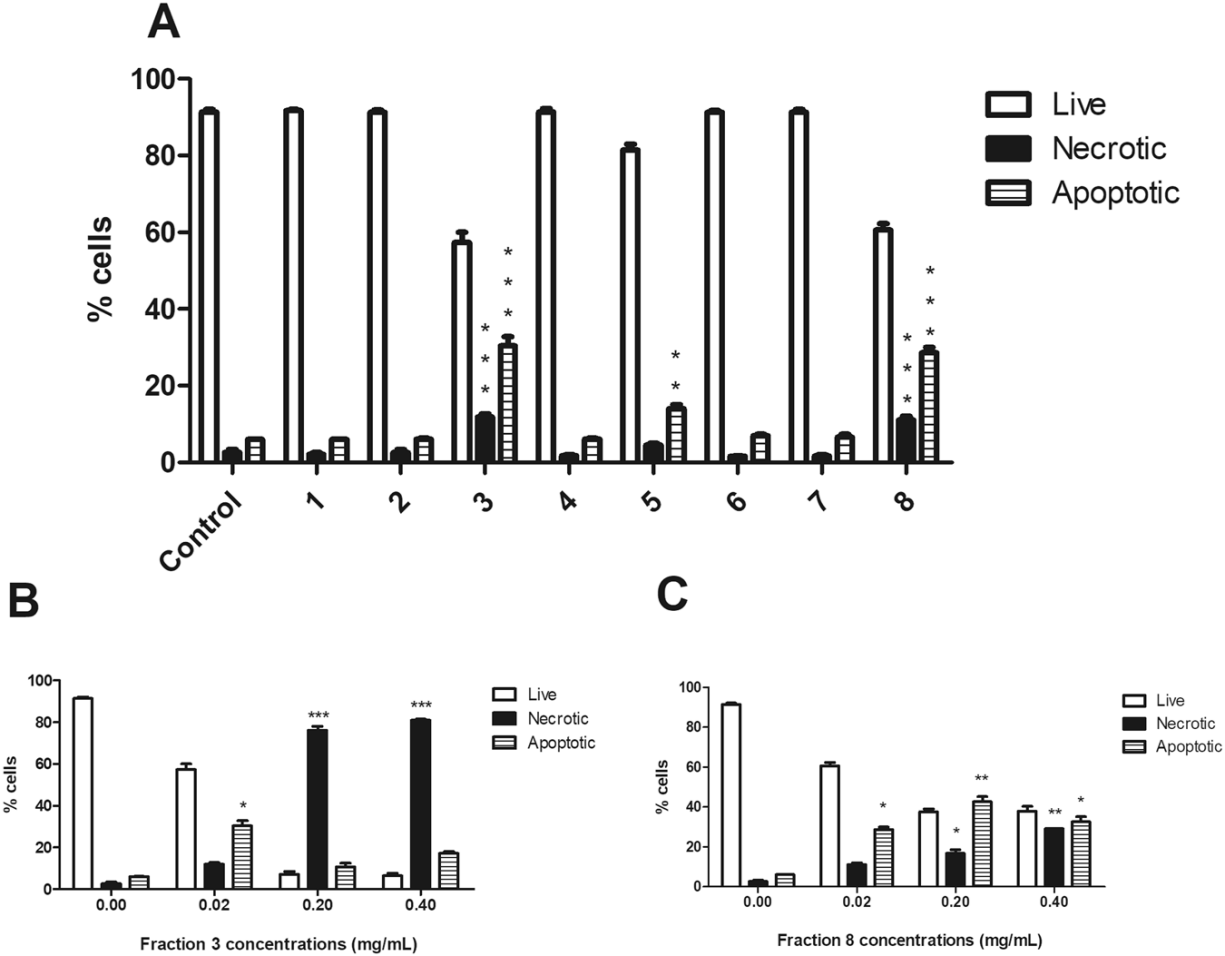
Cytotoxic effects induced by the different fractions of *H*. *indicus* decoction on Jurkat cells. Percentage of live, necrotic and apoptotic cells after 24 h Jurkat treatment at the lowest tested concentration (0.02 mg/mL) (**A**). Percentage of live, necrotic and apoptotic cells after 24 h Jurkat treatment with increasing concentrations of fraction 3 (**B**) or 8 (**C**). Data are the mean of three different experiments. *P < 0.05, **P < 0.01, ***P < 0.001 *versus* untreated cells.

We tested the pro-apoptotic activity of the eight fractions obtained from the lipophilic part of HI decoction. After 24 h from treatment, fractions 3 and 8 resulted the most cytotoxic: at the lowest tested concentration (0.02 mg/mL), the percentage of apoptotic cells was about 30% for both fractions, compared to 6% in untreated cells (Fig. 1A). At the highest concentrations, fraction 3 showed a significant increase in necrotic events, which markedly exceeded both viable and apoptotic cells (Fig. 1B). At the highest tested concentration, fraction 8 induced a 30% of both apoptotic and necrotic cells (Fig. 1C).

The analysis of the chemical composition and cytotoxicity of the decoction and its eight fractions indicated the presence of 3,4A, 2,4A and 2,4Acid in the most cytotoxic fractions of HI decoction. For this reason, we used 3,4 A, 2,4A and 2,4Acid as marker compounds for the decoction of HI, as indicated in the guidelines for Botanical Drug Development issued by the Food and Drug Administration^15^.

### HI and its main phytomarkers decrease cell viability

**Figure 2:**
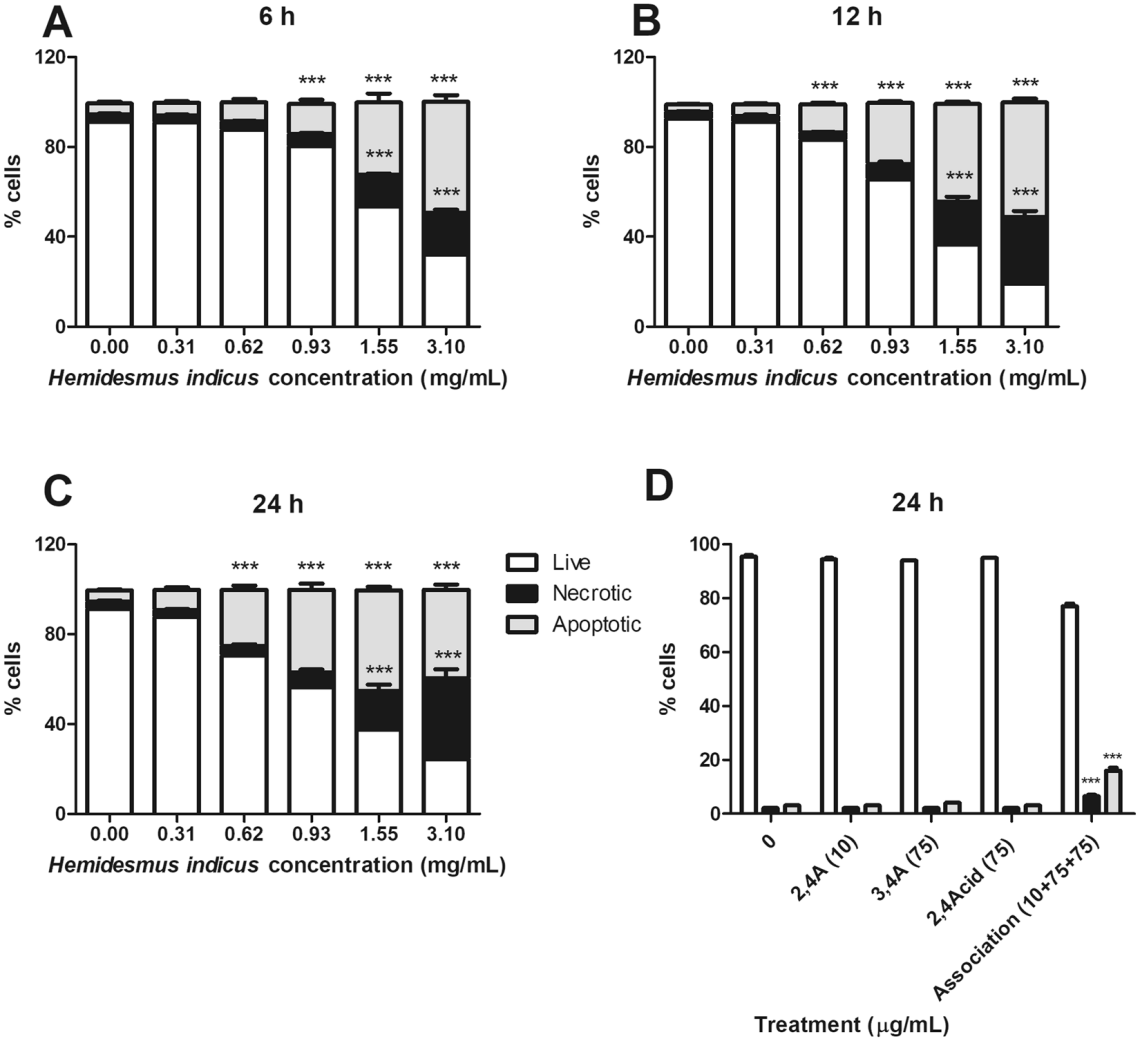
Cytotoxic effects induced by *H*. *indicus* and its phytomarkers on Jurkat cells. Percentage of live, necrotic and apoptotic cells after Jurkat treatment with increasing concentrations of *H*. *indicus* decoction (0.31–3.10 mg/mL) for (**A**) 6, (**B**) 12 or (**C**) 24 h. Data are the mean of at least six different experiments. Percentage of live, necrotic and apoptotic cells after Jurkat treatment with 2,4 A, 3,4 A and 2,4Acid or their association for 24 h (**D**). Data are the mean of three different experiments. **P < 0.01, ***P < 0.001 *versus* untreated cells.

HI decreased cell viability of Jurkat cells at all concentrations and times of treatment. After 6 h, the fraction of apoptotic cells was significant starting from 0.93 mg/mL HI (Fig. 2A). At 12 h, the percentage of apoptotic cells increased starting from 0.62 mg/mL (Fig. 2B). The same effect was observed after 24 h of treatment (Fig. 2C). At the highest tested concentrations and at all tested time points, we recorded a significant increase in necrotic events (Fig. 2A–C). To exclude the conditions in which the fraction of necrotic cells exceeded the viable and the apoptotic cells, we used concentrations up to 1.55 mg/mL at 12 and 24 h.

After treatment for 24 h with the association of 2,4A (2.5, 5.0 and 10 µg/mL), 3,4A (25, 50 and 75 µg/mL) and 2,4Acid (25, 50 and 75 µg/mL) (Supplementary Fig. 1), an increase in apoptosis was observed only for the association and at the highest tested concentrations (Fig. 2D). Of note, those concentrations are at least three times higher than their concentration in the decoction.

### HI increases intracellular levels of reactive oxygen species (ROS)

**Figure 3:**
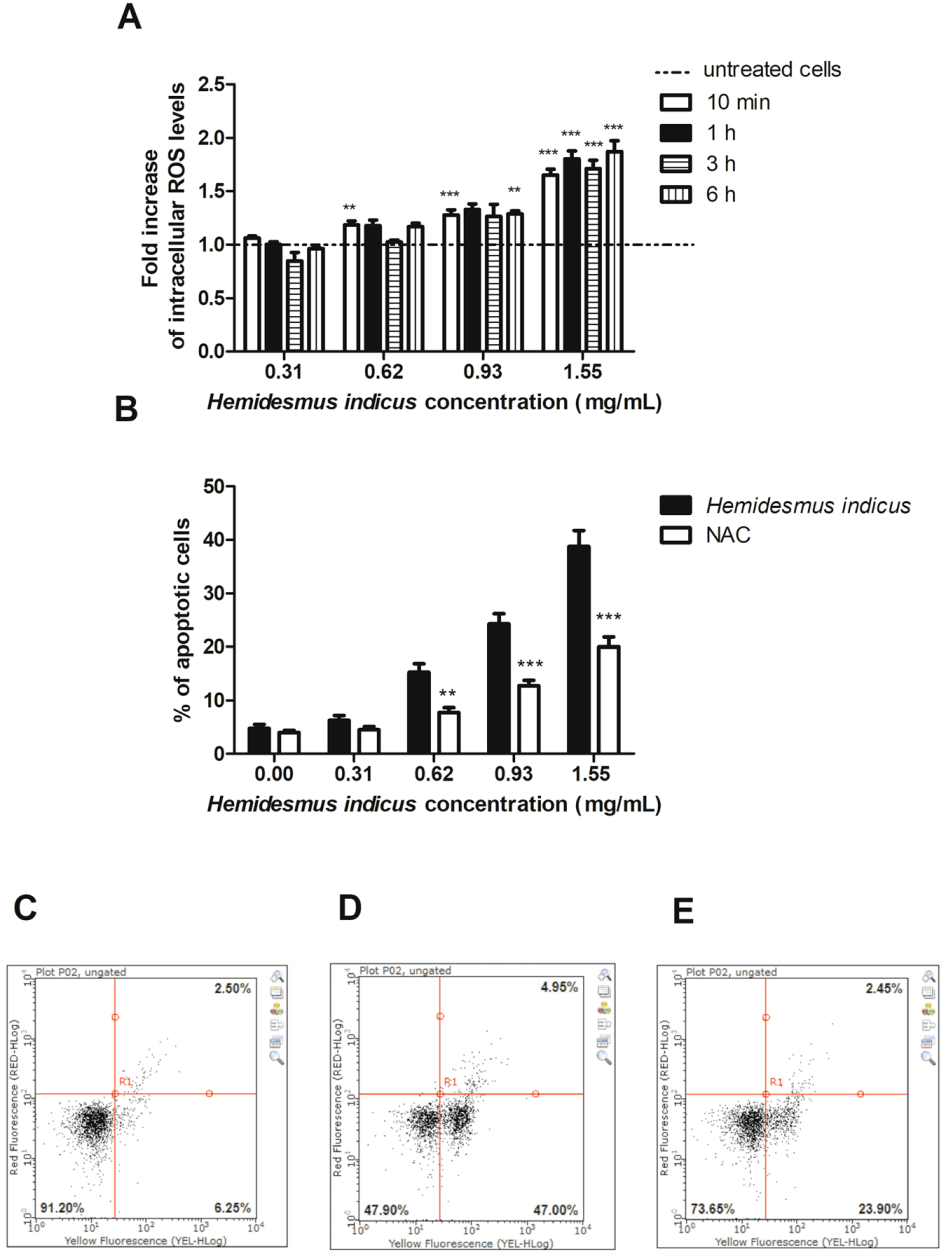
ROS relative levels after treatment of Jurkat cells for 10 min, 1, 3 or 6 h with *H*. *indicus* (**A**). Percentage of apoptotic cells after 24 h treatment with *H*. *indicus* in absence or presence of NAC 10 mM (**B**). Data are the mean of at least three different experiments. Representative plots of cells untreated (**C**) and treated with *H*. *indicus* 1.55 mg/mL in absence (**D**) or presence (**E**) of NAC. The percentage in lower/left quarter represents live cells, lower/right quarter apoptotic cells and upper/right quarter necrotic ones according to Guava Nexin assay. **P < 0.01, ***P < 0.001 *versus* untreated cells (dashed line in ROS level graph).

Treatment with HI increased intracellular ROS levels (Fig. 3A). In particular, after 6 h from treatment at the concentration 0.93 mg/mL the increase in ROS levels was 1.29 fold and reached a 1.87 fold-increase at the highest tested concentration. In order to assess the role of ROS in the pro-apoptotic activity of HI, we pre-treated cells with N-acetylcysteine (NAC), which is able to increase the intracellular GSH levels. The results clearly showed a significant reduction of the pro-apoptotic activity of HI (Fig. 3B–E).

### HI differently regulates the expression of pro-apoptotic genes

**Figure 4:**
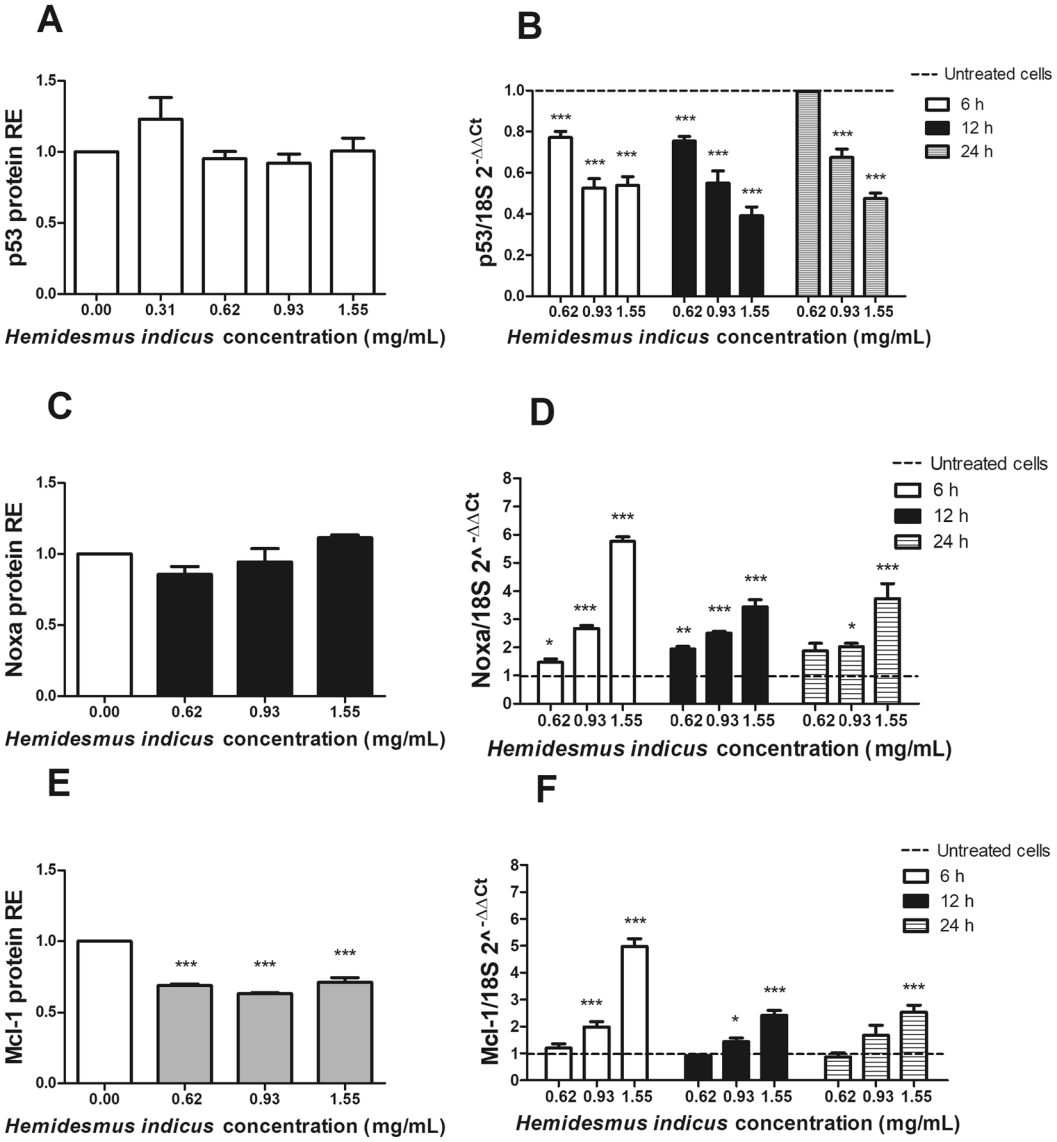
p53, Noxa and Mcl-1 expression after *H*. *indicus* treatment at protein and gene levels. Relative expression (RE) of (**A**) p53, (**C**) Noxa and (**E**) Mcl-1 protein after 24 h from *H*. *indicus* treatment. Modulation of (**B**) p53, (**D**) Noxa and (**F**) Mcl-1 mRNA following 6, 12 or 24 h culture with *H*. *indicus*. Data are the mean of at least three experiments. *P < 0.05, **P < 0.01 and ***P < 0.001 *versus* untreated cells (dashed line in mRNA expression graphs).

In order to define the mechanism of the pro-apoptotic activity of HI, we analyzed the expression level of crucial genes involved in the regulation of the apoptotic pathway. We first studied the protein and mRNA expression of p53. HI did not induce any significant variation in the protein expression of p53 (Fig. 4A), while showed a clear dose-dependent down-regulation of p53 mRNA (Fig. 4B).

The study of the pro- and anti-apoptotic proteins modulated by HI continued analyzing its ability to affect the expression of Noxa and Mcl-1 and their ratio. HI did not induce any modulation of Noxa expression (Fig. 4C), but it induced a significant dose-dependent up-regulation at RNA level (Fig. 4D). We recorded the highest expression of Noxa after 6 h and 1.55 mg/mL (5.77 ± 0.16). Furthermore, HI reduced the protein expression of Mcl-1 at all tested concentrations and time points (Fig. 4E). On the other hand, the RNA expression of Mcl-1 resulted up-regulated at the highest tested concentrations (Fig. 4F). The highest effect was recorded after 6 h at 1.55 mg/mL (4.98 ± 0.28). The Noxa/Mcl-1 proteins ratio, calculated using the relative expression of both proteins normalized to the untreated sample after 24 h-treatment with HI, was significantly higher compared to untreated cells (data not shown) and reached the highest value at 1.55 mg/mL (1.60 ± 0.09).

**Figure 5:**
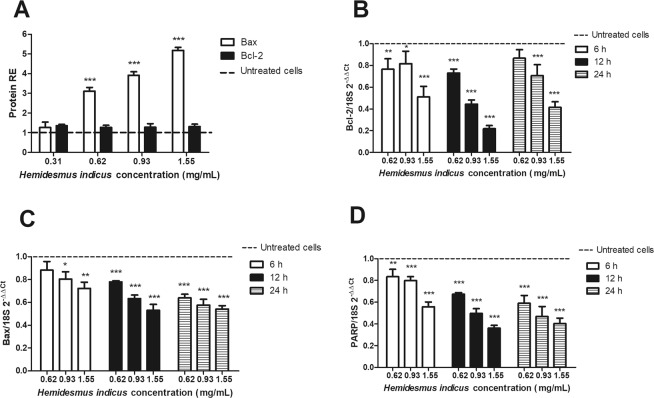
Relative expression (RE) of apoptotic proteins and genes after *H*. *indicus* treatment. (**A**) Bax or Bcl-2 protein RE after treatment of Jurkat cells with *H*. *indicus* for 24 h. (**B**) Bcl-2, (**C**) Bax, and (**D**) PARP mRNA expression after 6, 12 and 24 h from *H*. *indicus* treatment. Data are the mean of at least three experiments. *P < 0.05, **P < 0.01 and ***P < 0.001 *versus* untreated cells (dashed line).

Moreover, HI significantly increased the expression of Bax and slightly increased Bcl-2 protein levels (Fig. 5A). As regards its effect on Bax and Bcl-2 mRNA expression, both genes were down-regulated in a dose-dependent manner (Fig. 5B,C). We recorded similar effects for PARP: we previously reported that HI increased the level of cleaved PARP protein^13^; here, we observed that HI decreased its mRNA expression level (Fig. 5D).

### HI inhibits proteasome activity

**Figure 6:**
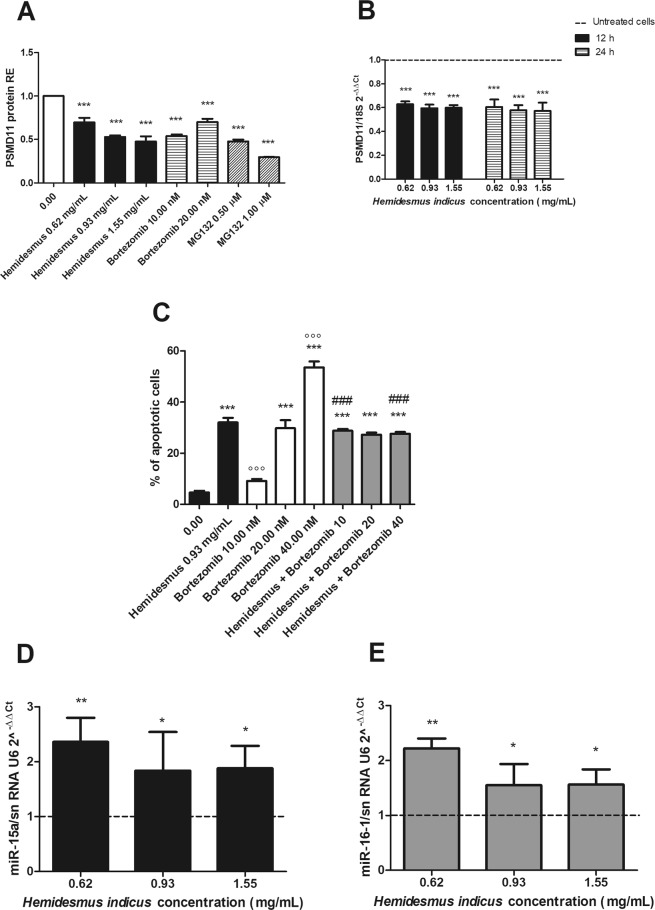
Post-transcriptional regulation after *H*. *indicus* treatment: proteasome expression, % of apoptotic cells after treatment with *H*. *indicus* plus bortezomib and upregulation of miR-15a and miR-16-1. (**A**) PSMD11 protein relative expression (RE) after treatment of Jurkat cells with *H*. *indicus*, bortezomib or MG132 for 24 h. (**B**) Reduction of PSMD11 mRNA expression following 12 or 24 h culture in the presence of *H*. *indicus*. (**C**) % of apoptotic cells after 24 h of Jurkat treatment with *H*. *indicus* 0.93 mg/mL, bortezomib or with the association of *H*. *indicus* plus bortezomib (scheme A). (**D**) Relative expression (RE) of miR-15a and (**E**) miR-16-1 after 24 h from *H*. *indicus* treatment of Jurkat cells. Data are the mean of at least three experiments. *P < 0.05; **P < 0.01; ***P < 0.001 *versus* untreated cells (dashed line in mRNA and miRNAs expression graphs); ^°°°^P < 0.001 *versus H*. *indicus*; ^###^P < 0.001 *versus* bortezomib.

Based on the different regulation observed after HI treatment at mRNA and protein levels for key genes involved in the apoptotic pathway, our next goal was to explore its potential ability to inhibit proteasome. The treatment of cells with HI induced a significant reduction in the protein expression of the 26 S proteasome (PSMD11) (Fig. 6A), which reached the highest down-regulation at the highest tested concentration (1.55 mg/mL, 0.48 ± 0.06). The proteasome inhibition of HI resulted comparable with that of bortezomib (0.54 ± 0.02) and MG132 (0.23 ± 0.01), used as reference proteasome inhibitors. Furthermore, HI down-regulated the mRNA levels of PSMD11 (Fig. 6B), both after 12 and 24 h from treatment.

Based on the proteasome inhibitory activity of HI, we tested whether it could potentiate the pro-apoptotic effect of bortezomib. After treatment with bortezomib for 24 h, we observed a dose-dependent increase in the apoptotic events (9.2, 29.8 and 53.6% at 10, 20 and 40 nM, respectively) (Fig. 6C). The apoptosis induced by HI 0.93 mg/mL plus bortezomib (scheme A) is about 30% at all the doses of bortezomib tested (Fig. 6C). The calculated CI was >1, suggesting an antagonistic effect.

When cells were pre-treated with HI for 10 h and then with bortezomib until the end of treatment (24 h) (scheme B), the results were similar to those recorded in the scheme A (Supplementary Fig. 2). On the other hand, the pre-treatment of cells with bortezomib for 10 h and then with HI until the end of treatment (24 h) (scheme C) decreases the percentage of apoptotic events compared to bortezomib alone. In particular, at the lowest tested concentration (10 nM bortezomib + 0.93 mg/mL HI), the percentage of apoptotic cells was similar to the treatment with bortezomib alone, whereas at increasing concentrations of bortezomib (20 and 40 nM), the apoptotic events were lower than bortezomib alone (Supplementary Fig. 2). For instance, at 40 nM bortezomib, the apoptosis recorded was 53.6%; after co-treatment with HI, the apoptotic events fall down to 31.2%, similar to the treatment with HI alone, confirming the antagonistic mechanism between the two drugs.

### HI up-regulates the expression of miR-15a and miR-16-1

We explored the expression of miR-15a and miR-16-1, two well-known tumor-suppressor miRNAs associated with the regulation of the 3′-UTR region of Bcl-2^16^ and recorded the up-regulation of both miR-15a and miR-16-1 relative to control cells (Fig. 6D,E). In particular, after 24 h from Jurkat treatment, the expression of both miRNAs was significantly increased. At 0.62 mg/mL, we recorded an up-regulation of 2.4 ± 0.4 for miR-15a and of 2.2 ± 0.2 for miR-16-1.

### Intestinal absorption of HI’s phytomarkers

**Figure 7:**
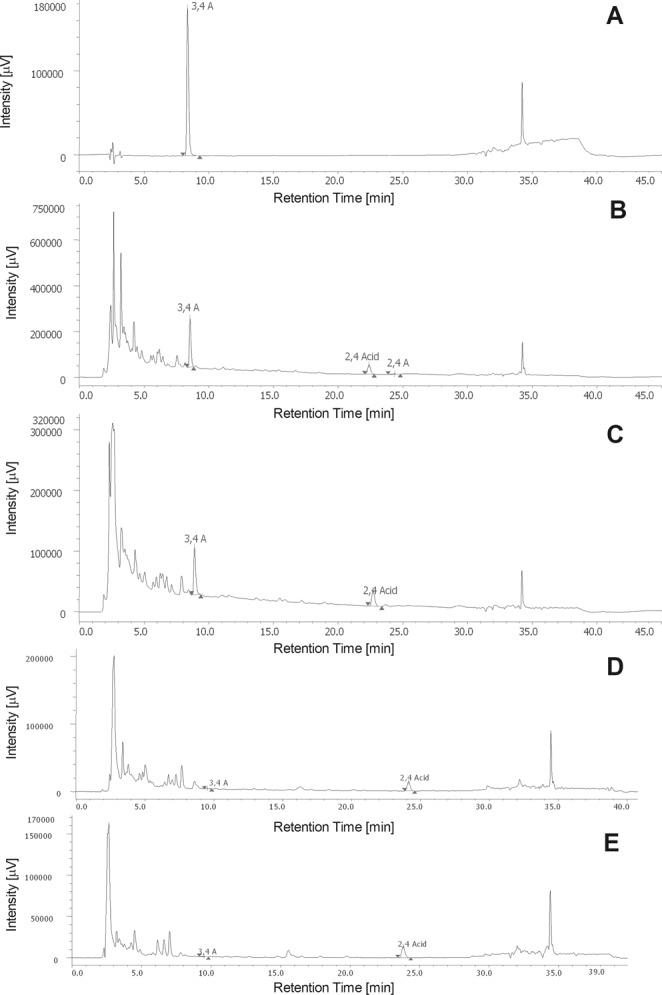
Chromatograms of (**A**) adducts between the aldehydes after 24 h of permanence in HBSS + 25 mM HEPES solution; (**B**) 2,4A, 3,4A and 2,4Acid detectable in *H*. *indicus* solution 31 mg/mL brought to dryness; (**C**) solution of BL chamber after 24 h-exposure to *H*. *indicus* 1.55 mg/mL; (**D**) BL solution after 24 h-exposure of A chamber to *H*. *indicus* 1.55 mg/mL; (**E**) A solution after 24 h-exposure of BL chamber to *H*. *indicus* 1.55 mg/mL.

We previously checked the monolayer integrity and excluded a cytotoxic effect of HI on differentiated enterocytes by FITC-dextran (data not shown). Before proceeding to the analysis of HI passage through the monolayer, we tested the ability of aldehydes to form adducts in HBSS + 25 mM HEPES. No adducts were detected after 24 h (Fig. 7A) or 48 h (data not shown). Furthermore, we excluded the possible degradation of aldehydes during the drying process of HI testing their presence in the stock solution of HI decoction after the drying process (Fig. 7B). Of note, the concentration of 2,4A in the stock solution resulted close to the HPLC detection limit.

In the second stage of our study, we put HI 1.55 mg/mL on the basolateral (BL) chamber, where we analyzed the presence of the three phytomarkers. Then, we put HI 1.55 mg/mL on the apical (A) chamber, where we analyzed the presence of the three phytomarkers. The HPLC analysis confirmed the presence of 3,4 A and 2,4Acid in both the BL (Fig. 7C) and the A chamber (data not shown) in the same proportion in which they are present in the stock solution. As expected, it was not possible to detect 2,4A.

In the next stage of our study, we tested whether the phytomarkers pass the enterocytes monolayer (Supplementary Fig. 3). After 24 h from the exposure of the A chamber to HI 1.55 mg/mL, we analyzed the BL compartment solution. The same experiments were performed to test the passage from the BL to the A compartment. In both cases, we recorded the acid, but it was more difficult to detect the aldehydes (Fig. 7D,E, respectively). We performed similar experiments at shorter times (3 h) of treatment of A or BL chamber with HI observing similar results (data not shown).

Once the experiments of monolayer passage were performed, the cells were lysed and analyzed for the presence of HI’s phytomarkers. We did not detect HI’s components (data not shown). This means that phytomarkers were not trapped into the enterocytes.

## Discussion

**Figure 8:**
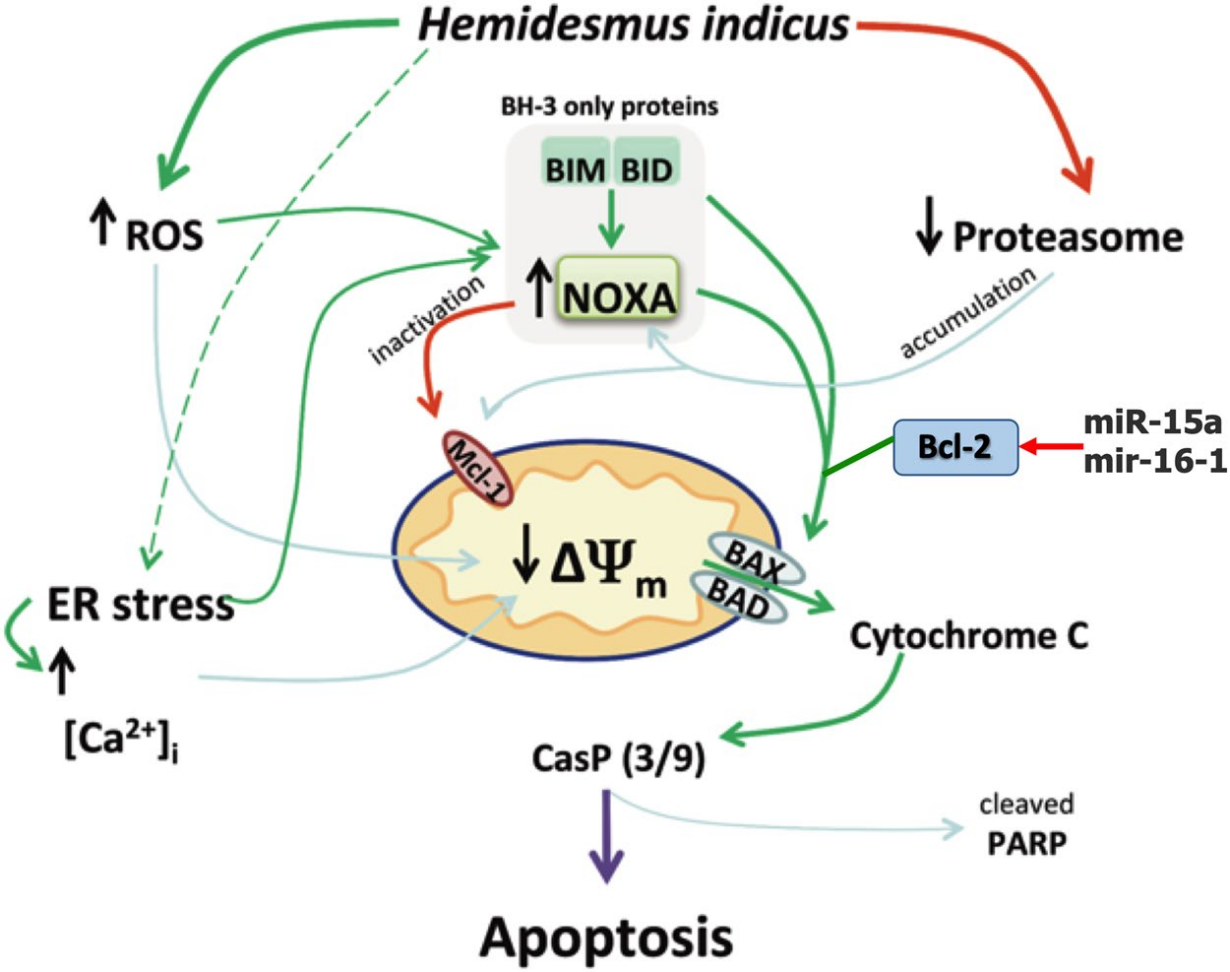
Molecular targets of *H*. *indicus*. ER: endoplasmic reticulum; [Ca^2^^+^]_i_: intracellular calcium; CasP: caspase; ΔΨ_m_: transmembrane mitochondrial potemtial.

In this paper, we report a potent antileukemic effect for HI, imputable to the modulation of different critical targets (Fig. 8) involved in the apoptotic pathway. In our previous^13^ and current results, we show that HI treatment markedly induced Bax and Bcl-2 protein expression and their ratio, suggesting that Bax was up-regulated and played an important role in the induction of apoptosis by HI. However, in the current study, we report that HI treatment reduced the mRNA expression of Bcl-2 and Bax. This result was apparently in conflict. Since an ubiquitin-proteasome dependent pathway degrades both proteins, we studied the ability of HI to inhibit this pathway.

The 26S proteasome is critical for the maintenance of homeostasis of most intracellular proteins^2^. Ubiquitinylation and proteasome-mediated degradation regulate a large number of proteins involved in cell cycle and apoptosis. Therefore, targeting proteasome pathway has emerged as a promising approach to cancer therapy^17^. In our experimental model, HI reduced the 26S proteasome expression. Its ability was comparable with that expressed by bortezomib and MG132. Furthermore, HI induced a post-transcriptional down-regulation of the proteasome mRNA. The proteasome inhibition activity of HI can partially justify the differential post-transcriptional regulation of Bax and Bcl-2 evoked by HI. In fact, HI decoction reduces, but does not suppress, the expression of Bax at mRNA level; this means that cells still produce the protein. Since HI inhibits the proteasome, treatment of cells with HI results in accumulation of proteins if compared with the untreated cells.

The different regulation at protein and gene level could be due to mechanisms acting at post-transcriptional level including miRNAs^18^. Our data show that HI induced a strong up-regulation of Bax protein levels and a slight up-regulation of Bcl-2 levels, which resulted in the up-regulation of Bax/Bcl-2 protein ratio. We recorded the up-regulation of miR-15a and miR-16-1, which are associated with the regulation of the 3′-UTR region of Bcl-2^16^. The increase in miR-15a/16-1 expression has been reported to increase Bax/Bcl-2 protein ratio^19^.

Among the pro-survival Bcl-2 family members, Mcl-1 is essential for the survival of multiple cell lineages in the adult^20–23^. Human cancers frequently over-express Mcl-1^24^, which represents a key factor in the resistance of leukemia to anti-cancer therapy^25^. Mcl-1 is localized to distinct mitochondrial sub-compartments, with differential functions that affect mitochondrial activity and integrity. Mcl-1 exerts its anti-apoptotic activity on the outer mitochondrial membrane, where it antagonizes Bax and Bak activation, maintaining mitochondrial integrity, and inhibits mitochondrial calcium signals following an apoptotic stimulus^26^. In contrast, Mcl-1 localized in the mitochondrial matrix is unable to inhibit apoptosis, but maintains normal inner mitochondrial membrane structure and promotes the assembly of ATP synthase oligomers; thereby, it facilitates mitochondrial homeostasis and supports mitochondrial bio-energetic function^27^. A down-regulation of Mcl-1 is often sufficient to promote apoptosis in leukemic cells, suggesting that Mcl-1 can be a potential therapeutic target in the treatment of several human leukemias^25^. To date, small-molecule Mcl-1 inhibitors are emerging for cancer treatment^28^. However, to our knowledge, no Mcl-1 specific inhibitors are in clinic. Our study revealed that HI induces a rapid decrease in Mcl-1 protein levels. This regulation appeared to be essentially post-translational because Mcl-1 mRNA was up-regulated at all treatment times. The increased gene expression of Mcl-1 may represent a mechanism of leukemic cells’ protection against the degradation of the anti-apoptotic protein induced by HI. At post-translational level, Mcl-1 can be cleaved by caspase-3. This leads to the removal of a large part of the N-terminus of Mcl-1, leaving the BH1-3 and the C-terminal transmembrane domains intact. The caspase-dependent cleavage of Mcl-1 suggests that the cleavage products become pro-apoptotic^29^. Since HI stimulates caspase-3 activity^13^, its induction may be responsible for the post-translational down-regulation of Mcl-1. Furthermore, Mcl-1 is subject to a rapid turnover through ubiquitin-dependent protein degradation by the 26S proteasome. One of E3 ligases of the proteasome, MULE/LASU1, is a BH3-only Hect E3-ligase, whose BH3 domain interacts with the hydrophobic BH3 binding pocket of Mcl-1 and not with other pro-survival Bcl-2 family members^30^. Noxa associates mainly with Mcl-1 and induces ubiquitin-proteasome-mediated degradation of Mcl-1^31^. In our study, despite the clear post-transcriptional stimulation of Noxa induced by HI, the protein levels were not modulated, even if the proteasome inhibition ability of HI should have led to an accumulation of the BH3-only family protein. Reasonable explanations for this phenomenon could be: (1) the lack of a specific antibody able to recognize the epitope of Noxa in dimer with Mcl-1^32^; (2) the really short half-life of Noxa protein (1–2 h)^33^; (3) the high physiological level of Noxa in Jurkat cells that could make the detection of alterations in protein expression difficult^34^. Noxa/Mcl-1 balance plays an important role in the intrinsic apoptotic pathway^34^. Treatments with HI caused a variation in the Noxa/Mcl-1 ratio in favor of Noxa showing how much the down-regulation of Mcl-1 and its close relation with Noxa are crucial for the pro-apoptotic activity exerted by HI.

Senescence is an established cellular pathway involved in all aspects of cancer biology and appears to be a major obstacle for cancer progression^35^. Distinct pathways control senescence, but in general, it is initiated by tumor suppressors like p53. Thus, without the loss of tumor suppressor genes like p53, cells expressing or even over-expressing oncogenes fail to become cancerous due to senescence^36^. Mcl-1 plays an important role in preventing chemotherapy-induced senescence in both a p53-dependent and -independent manner. Mcl-1-mediated inhibition of senescence can enhance tumor growth. Moreover, an increased p21 expression is observed in all cases where senescence can be induced^36^. This allows speculating that Mcl-1 down-regulation represents a key step not only in the induction of apoptosis but also in the inhibition of the cell-cycle progression by HI^13^. The evidence that HI did not show any modulation of p53 in our experimental setting supports this result.

Other key factors in the regulation of cellular apoptosis pathway are ROS, whose levels influence several proteins involved in the apoptotic and cell-cycle regulation. Mitochondria are source and target of ROS. As an example, the release of cytochrome c and then the activation of caspases are directly and indirectly regulated by ROS^37^. Furthermore, several studies reported that p21 expression during senescence is dependent on the production of ROS^38^. HI increased the production of ROS. The significant reduction of apoptotic events observed after NAC pre-treatment confirms the key role of ROS in the pro-apoptotic activity of HI.

Many proteasome inhibitors provoke apoptosis through the activation of endoplasmic reticulum stress^39^ and induce Noxa-dependent generation of ROS. ROS increase is the main responsible for the pro-apoptotic activity of bortezomib and leads to the disruption of the mitochondrial potential^40,41^. In particular, proteasome inhibition induces the accumulation of unfolded proteins in the endoplasmic reticulum lumen, which promotes the overproduction of ROS and leads to fatal cell stress^42^. Furthermore, studies investigating the relationship between oxidative stress and apoptosis demonstrated the key role of this cause-effect mechanism in the cytotoxic activity of MG132 on rat C6 glioma cells^43^. In this regard, recent papers report that HI decoction induces endoplasmic reticulum stress via PERK-orchestrated pathways in colorectal cancer cells^11^, increases intracellular ROS level, and disrupts mitochondrial potential^13^. Those results support the proteasome inhibition activity of HI.

However, the association of HI plus bortezomib yielded unexpected results under our experimental conditions: we found that HI antagonized or did not enhance the cytotoxic activity of bortezomib. *Ad hoc* studies should be designed to investigate the possible mechanism of this interaction. Based on our experimental design, we can hypothesize that bortezomib may completely inhibit the proteasome function. When the ubiquitin-proteasome system is completely inhibited by bortezomib, HI cannot potentiate the inhibitory activity of bortezomib on proteasome function.

In the light of the interesting pharmacological profile of HI, we explored the intestinal absorption of its main phytochemicals. Our phytochemical analysis identified three phytomarkers^13,44^, which could be used to preliminary test the intestinal absorption of HI. Of note, the three molecules induced apoptotic events on Jurkat cells, but only in association and at concentrations three times higher than that present in the decoction, supporting the notion that the pharmacological effects of HI are due to its complex nature. Considering these premises, we decided not to evaluate the membrane passage of the single molecules, but, as major phytomarkers of the decoction, we used them to evaluate the permeation of the total phytocomplex.

When cultured as a monolayer, Caco2 cells differentiate and form tight junctions. In this way, they mimic the human intestinal epithelium and paracellular movement of drugs across the monolayer. Moreover, they express different proteins including transporter proteins, efflux proteins, and phase-2 enzymes^45^. We added the decoction of HI to either the A or BL chamber sides of the monolayer and, after treatment for different times, removed aliquots of solution in the A or BL chamber to measure the concentration of the three phytomarkers. We found that the acid passed through the monolayer. For the two aldehydes, the results are not clear. The difficulties to reveal aldehydes could be imputable to their air sensitivity^46^. Before chromatographic analysis, the samples were dried. This procedure allows their concentration but can favor their disappearance. Taking into account that we did not reveal the 2,4A and 3,4A after 24 h of enterocytes monolayer exposure, we may also hypothesize that the disappearance of the two molecules could be due to their metabolism^47^. Indeed, differentiated Caco2 cells exhibit a high expression level of cytochrome P450 enzymes and phase II enzymes (*i*.*e*. aldehyde dehydrogenase, UDP-glucuronosyltransferases, sulfotransferases, and glutathione-S-transferases)^48,49^.

In an attempt to extrapolate our data on intestinal absorption to peripheral blood, we may postulate that a part of aldehydes of HI will be metabolized in the liver by aldehyde dehydrogenase^50^, thus reducing the amount of aldehydes able to reach the blood. However, the isovanillic acid produced by the aldehyde dehydrogenase-catalyzed metabolism of aldehydes is endowed with cytostatic effects^51^ and can thus contribute to the pharmacological activity of HI. As regards 2,4Acid, it was recorded in rat plasma following oral administration^52^. Moreover, some *in vivo* studies reported its ability to affect plasma glucose, insulin, total hemoglobin, glycosylated hemoglobin, serum enzymes, and erythrocyte membrane bound enzymes following oral administration^53,54^. Taken together, those data support the hypothesis that 2,4Acid is able to reach the blood.

On the whole, our study supports the pharmacological potential of HI, which can represent an interesting botanical drug in the oncological area. Further studies are necessary to underline its *in vivo* molecular mechanisms and elucidate its clinical potential.

## Materials and Methods

### Plant decoction preparation and characterization

The plant (voucher #MAPL/20/178) was provided by MAP Italia [Caldiero (VR), Italy]. The plant was collected from Ram Bagh in Rajastan, India, authenticated by Dr. MR Uniyal, Maharishi Ayurveda Product Ltd (Noida, India), and prepared as previously described^13^. Our previous HPLC analyses demonstrated and quantified the presence of three main phytomarkers (2,4A, 3,4A and 2,4Acid) in the decoction^13^. In this paper, we applied the same method of analysis. After the preparation using the method mentioned above and its cooling, we fractionated the decoction into two parts using a dropping funnel with a mixture of chloroform/methanol/ethyl acetate (13:1:2). The lipophilic part was evaporated to dryness^55^. The dried material was then dissolved in the same solvent system and the main compounds of HI decoction were purified by chromatographic column with SiO_2_ as stationary phase and chloroform/methanol/ethyl acetate (13:1:2) as mobile phase. The stationary phase was then washed with 2 bed volume of methanol to elute the more polar compounds still trapped in the gravimetric column and obtain the last fraction at medium polarity (M). Eight main fractions were collected (1, 2, 3, 4, 5, 6, 7 and 8) and evaporated to dryness. We used pre-coated silica gel plates (HPTLC silica gel 60 F254; thickness 0.25 mm; Merck Millipore, Darmstadt, Germany) with the same above mobile phase to check the fraction separations. To analyze the less volatile molecules contained in the various fractions, an aliquot of few milligrams of each fraction was mixed with 200 μL of pyridine and 200 μL of BSTFA (1% TMCS) (bis(trimethylsilyl)trifluoroacetamide + trimethylchlorosilane) (Sigma, St. Louis, MO, USA), and heated for 60 min at 80 °C with the purpose of obtaining the trimethylsilylethers (TMS). We analyzed each obtained fraction by gas chromatography coupled with mass spectrometer (GC-MS). The analyses were performed by a Varian GC-3800 gas chromatograph (Palo Alto, CA, USA) equipped with a Varian MS-4000 mass spectrometer (Palo Alto, CA, USA) using electron impact and hooked to NIST library. The gas chromatographic conditions were as follows: injector temperature 300 °C, carrier (Helium) flow rate 1.2 mL/min, split ratio 1:50. The initial oven temperature was 100 °C, then raised to 320 °C at a rate of 5 °C/min, and then kept constant for 20 min. One microliter of each fraction was dissolved in CHCl_3_ (Sigma) and injected. The mass spectrometry conditions were the same reported in Guerrini *et al*.^56^.

### Cell culture and treatment

We obtained Jurkat T-lymphoblastic leukemia cells and Caco2 colon rectal cancer cells were obtained from ATCC cell bank (LGC Group, Middelsex, UK) and tested, characterized and authenticated by the cell bank for the species origin. Cells were propagated in RPMI 1640 or DMEM, respectively, supplemented with 10% heat-inactivated bovine serum, 1% penicillin/streptomycin solution, and 1% L-glutamine solution (all obtained from Sigma). Cells were treated with different concentrations of HI (0.31–3.10 mg/mL) obtained from an aqueous stock solution (31 mg/mL) or with its main phytomarkers at concentrations equal or three-times higher than those of the decoction. In some experiments, cells were treated with a DMSO solution of bortezomib (2.50–40.00 nM; Selleckchem, Houston, TX, USA) or MG132 (0.10–5.00 μM; Sigma) or pre-treated with NAC, 1 h, 10 mM; Sigma).

### Flow cytometry

All flow cytometric analyses were performed using an EasyCyte 5HT flow cytometer (Guava Technologies-Millipore, Hayward, CA, USA).

### Analysis of apoptosis

We analyzed apoptosis for the eight fractions obtained from the lipophilic part of the decoction. Each fraction was dissolved in DMSO and tested at different concentrations (0.02–0.40 mg/mL). Exposure of cell cultures to 1% DMSO had no significant effect on cell viability. Thus, 1% was the highest tested concentration of each fraction. We analyzed apoptosis also after the following treatments with the decoction: (1) HI at 6, 12 or 24 h from treatment; (2) bortezomib or MG132 at 24 h from treatment; (3) HI (0.93 mg/mL) plus bortezomib (10.00–40.00 nM) at 24 h from treatment. In the treatment indicated as 3, we used three different experimental strategies: scheme A: co-treatment of cells with HI + bortezomib; scheme B: treatment with HI for 10 h and then treatment with bortezomib; scheme C: treatment with bortezomib for 10 h and then treatment with HI. Aliquots of 2 × 10^4^ cells were stained with 100 µL of Guava Nexin Reagent (Merck Millipore) containing Annexin V-phycoerythrin (Annexin V-PE) and 7-amino-actinomycin D (7-AAD) and analyzed via flow cytometry.

### Total RNA isolation

Extraction of total RNA was performed by mirVana^TM^ miRNA Isolation Kit (Ambion, Austin, TX, USA) after treatment for 6, 12 or 24 h with HI, or after 24 h with bortezomib (10 and 20 nM) or MG132 (0.50 and 1.00 µM). Cells were treated with lysis buffer, then subjected to acid-phenol:chloroform extraction. Ethanol was added to samples and then samples were passed through a filter cartridge containing a glass-fiber filter, which immobilizes the RNA. The filter was washed and finally RNA was eluted with a low ionic strength solution. The RNA collected was stored at −20 °C.

RNA integrity analysis was performed by microfluidic capillary electrophoresis with the Agilent 2100 bioanalyzer (Agilent Technologies, Palo Alto, CA, USA) to guarantee the good quality of analyzed RNA^57^.

### cDNA synthesis

Total RNA was reverse transcribed using High-Capacity cDNA Reverse Transcription kit (Life Technologies, Carlsbad, CA, USA). Briefly, 200 ng total RNA were added to 10 µL reaction kit mixture containing an RNase inhibitor and subjected to thermal cycler conditions according to the manufacturer’s recommendation. The obtained cDNA was stored at −20 °C.

### mRNA analysis

Quantification of Bax, Bcl-2, PARP, p53, PSMD11, Noxa (PMAIP1) and Mcl-1 mRNA as well as 18 S rRNA as internal control was performed by real-time PCR (ABI Prism 7900 HT, Life Technologies) using Universal Master Mix (Life Technologies) and TaqMan assays (Hs00180269_m1, Hs00608023_m1, Hs00234387_m1, Hs01034249_m1, Hs00160660_m1, Hs00560402_m1, Hs01050896_m1, Hs99999901_s1, respectively).

### miRNA analysis

miRNA expression pattern was determined by Low Density Arrays (TaqMan® array Human MicroRNA card A and B v3.0) (Life Technologies) that allow the screening of 754 miRNAs. miRNAs were reverse transcribed from 800 ng of total RNA using Megaplex™ Primer Pools, Human Pools A and B v3.0 and TaqMan® MicroRNA Reverse Transcription kit (Life Technologies). After adding the Universal Master Mix (Life Technologies), cDNA was loaded on the arrays in a volume of 100 µL per port. For validation, 800 ng of total RNA were again reverse transcribed applying RT primers specific for the studied miRNAs and using snRNA U6 and RNU48 as endogenous controls in a triplex approach. cDNA quantification was performed using Universal Master Mix (Life Technologies) and TaqMan®MicroRNA individual assays for miR-15a and miR-16-1 (ID 000389 and ID 000391, respectively), snRNA U6 (ID 001973) and RNU48 (ID 001006) (Life Technologies) in singleplex PCR reactions.

All described qRT-PCR reactions were performed on an ABI Prism 7900HT (Life Technologies). Relative expression was calculated using the 2^−ΔΔCt^ quantification method^58^, which allows the final result to be presented as the fold change of target gene expression in a target sample relative to a reference sample, normalized to a reference gene.

### Analysis of apoptotic proteins

After treatment with HI for 24 h, samples were analyzed for p53, PSMD11, Noxa, Mcl-1, Bax, Bcl-2 protein expression. Additionally, PSMD11 expression was studied in cells treated for 24 h with bortezomib or MG132. Briefly, 1 × 10^6^ cells were fixed and permeabilized and then incubated with the following antibodies: p53 [20 µL, Alexa Fluor® 488 Mouse anti-p53 (pS37), BD Biosciences (Franklin Lakes, NJ, USA)], PSMD11 [1:100, mouse monoclonal (AT1F4) anti-PSMD11, Abcam (Cambridge, United Kingdom)], Noxa [2:100, mouse monoclonal antibody (1144C307) anti-Noxa, Abcam], Mcl-1 (1:100, mouse monoclonal IgG1, Abcam), Bax [1:100, mouse monoclonal IgG1 (sc-20067), Santa Cruz Biotechnology (Santa Cruz, CA, USA)] Bcl-2 [1:100, mouse monoclonal IgG1 (sc-509), Santa Cruz Biotechnology]. The samples were washed and incubated, except p53, with the secondary fluorescein isothiocyanate (FITC) anti-mouse IgG (1:100, Sigma), and analyzed via flow cytometry. Mean fluorescence intensity values were calculated.

### Measurement of ROS

To determine the generation of intracellular ROS, the dichlorofluorescein assay was used after 10 min, 1, 3 or 6 h of treatment with HI. Briefly, 1 × 10^6^ cells were washed with PBS 1x and incubated with 10 µM 2′,7′–dichlorodihydrofluoresceine diacetate (H2-DCFDA) (Sigma) for 20 min at 37 °C and 5% CO_2_. Cells were washed and measured flow cytometrically for oxidation of H2-DCFDA.

### Study of intestinal absorption in Caco2 monolayers

10^5^ Caco2 cells were seeded in monolayer on a polycarbonate insert (Millicel, Merck Millipore) with a 12 mm diameter and a 4 µm pore dimension. After 21 days to complete cell differentiation in enterocytes, the support allows distinguishing an A chamber and a BL one, in connection through the monolayer of cells. Chamber A contains 400 µL complete medium and chamber BL 600 µL. To test the monolayer integrity, FITC-dextran 70 and 4 KDa were used. A solution of 2.5 mg/mL FITC-dextran in HBSS + HEPES 25 mM was added to A chamber and after 20 min aliquots of BL chamber solution were taken for the analysis of fluorescence at TECAN spectrophotometer (TECAN, Zurich, Switzerland). The analysis was performed after 30 min and 1, 2, 3, 4, 6 and 24 h. Using the same protocol, the passage of FITC-dextran was analyzed in the opposite direction, BL *versus* A. In both experimental systems, the recorded fluorescence is proportional to the dextran permeated through the enterocyte monolayer. Once the integrity of the monolayer was checked, HI 1.55 mg/mL was put on A chamber to analyze the passage to BL chamber or *vice versa* (Supplementary Fig. 3). The BL and A solutions, respectively, were collected after 3 and 24 h from the monolayer exposure to HI. The liquid was removed under vacuum to concentrate the substances passed through the monolayer and the resulting residue was analyzed via chromatography to verify the presence of the main phytomarkers contained in the decoction: 2,4A, 3,4A and 2,4Acid.

The integrity of the monolayer was checked also after HI treatment to verify that the decoction did not exert cytotoxic effects on enterocytes. Furthermore, to investigate whether HI components were trapped into intracellular compartments, at the end of the experiment the monolayer was collected and lysated by sonication. Samples were concentrated and analyzed for the presence of the main HI’s phytomarkers.

### Statistical analysis

All results are expressed as mean ± SEM. Differences among treatments were evaluated by repeated ANOVA, followed by Bonferroni or Dunnett as post-test, using GraphPad InStat version 5.00 (GraphPad Prism, San Diego, CA, USA). P < 0.05 was considered significant. Interaction between HI and bortezomib was classified using the combination index approach^59^.

## Supplementary information

Dataset 1
