# Supplementary material for: Hemidesmus indicus induces apoptosis via proteasome inhibition and generation of reactive oxygen species
Source: Sci Rep. 2019 May 10;9:7199. doi: 10.1038/s41598-019-43609-5 (PMC6510901; doi:10.1038/s41598-019-43609-5)
Supplement: Supplementary file 1 — Dataset 1 [file 41598_2019_43609_MOESM1_ESM.docx]

***Hemidesmus indicus* induces apoptosis via proteasome inhibition and ROS generation**

**Eleonora Turrini^1^, Elena Catanzaro^1^, Lorenzo Ferruzzi^1^, Alessandra Guerrini^2^, Massimo Tacchini^2^, Gianni Sacchetti^2^, Guglielmo Paganetto^2^, Francesca Maffei^1^, Valentina Pellicioni^1^, Ferruccio Poli^3^, Patrizia Hrelia^3^, Manuela Mandrone^3^, Piero Sestili^4^, Maurizio Brigotti^5^, Carmela Fimognari^1^,***

^1^Department for Life Quality Studies, University of Bologna, Rimini, Italy

^2^Department of Life Sciences and Biotechnology, University of Ferrara, Malborghetto di Boara, Ferrara, Italy

^3^Department of Pharmacy and Biotechnology, University of Bologna, Bologna, Italy

^4^Department of Biomolecular Sciences, University of Urbino "Carlo Bo", Urbino, Italy

^5^Department of Experimental, Diagnostic and Specialty Medicine, University of Bologna, Bologna, Italy

*To whom correspondence should be addressed. Tel: +39 0541 434658; Email: [carmela.fimognari@unibo.it](mailto:carmela.fimognari@unibo.it) (ORCID: 0000-0002-2461-8214)


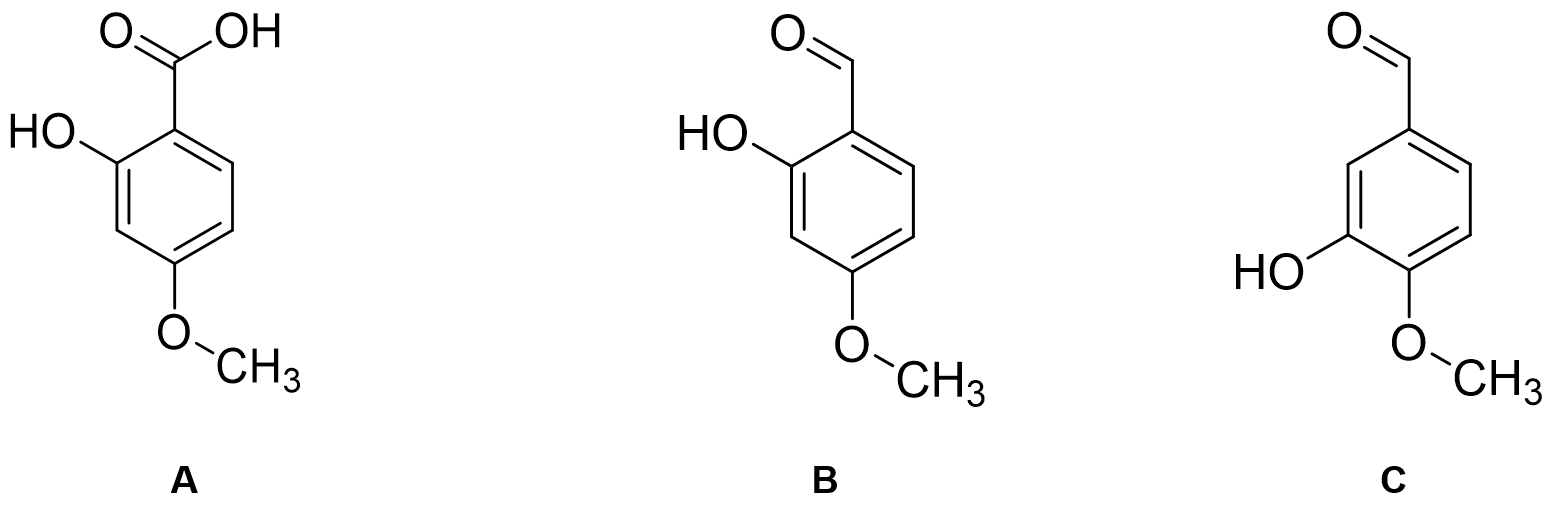


**Supplementary Fig. 1.** 2-hydroxy 4-methoxybenzoic acid (2,4Acid) (**A**), 2-hydroxy 4-methoxybenzaldehyde (2,4A) (**B**), 3-hydroxy 4-methoxybenzaldehyde (3,4A) (**C**).


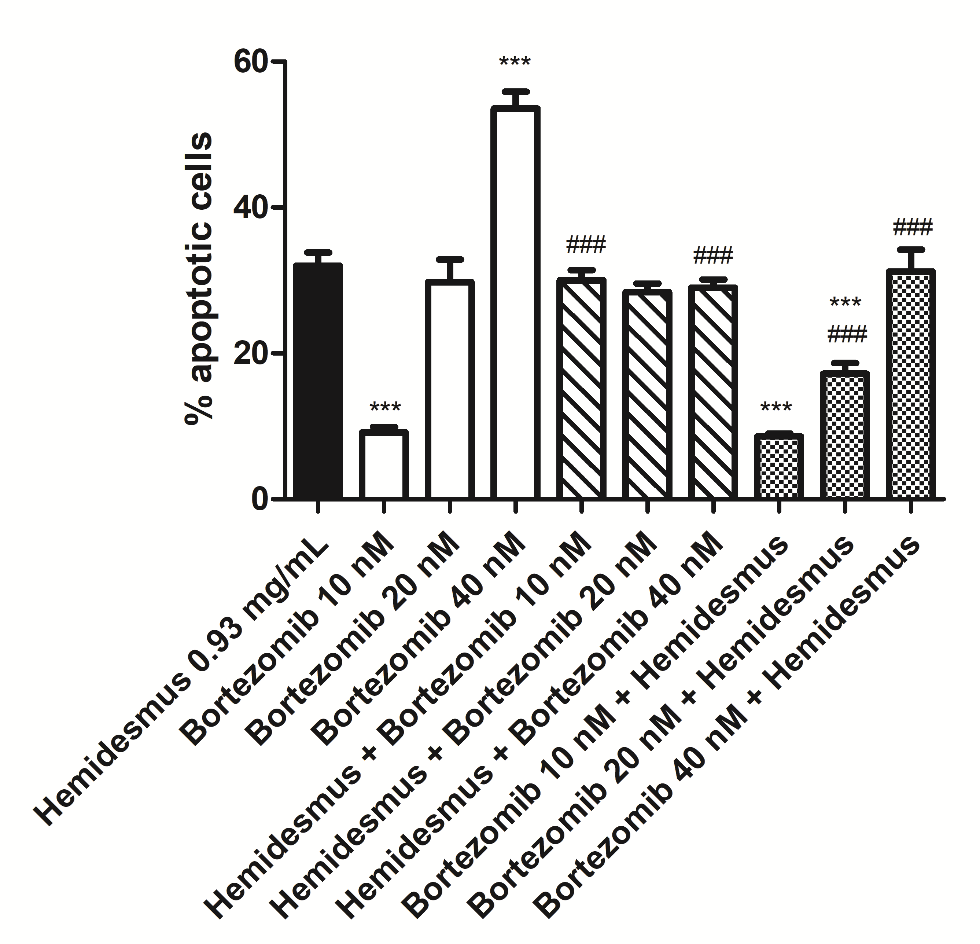


**Supplementary Fig. 2.** Apoptotic cells after treatment with *H. indicus* plus bortezomib. % of apoptotic cells after 24 h of Jurkat treatment with *H. indicus* 0.93 mg/mL, bortezomib or with the association of *H. indicus* plus bortezomib, according to scheme B or scheme C. Data are the mean of at least three independent experiments. ***P < 0.001 *versus H. indicus*; ###P < 0.001 *versus* bortezomib.


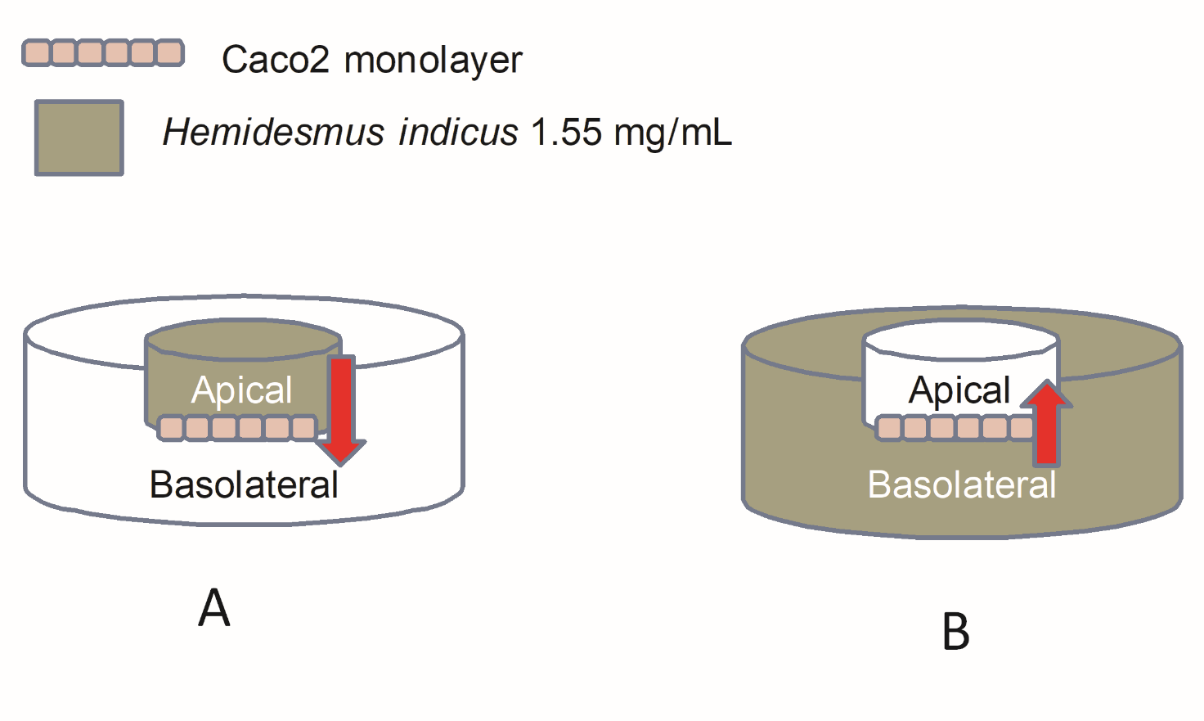


**Supplementary Fig. 3.** Schematic representation of the experiments performed on Caco2 monolayers. After 3 or 24 h, the passage of *H. indicus’* phytomarkers from Apical to Basolateral chamber (**A**) or *vice versa* (**B)** was analyzed.
